# RhoA GTPase Switch Controls Cx43-Hemichannel Activity through the Contractile System

**DOI:** 10.1371/journal.pone.0042074

**Published:** 2012-07-30

**Authors:** Raf Ponsaerts, Catheleyne D’hondt, Fréderic Hertens, Jan B. Parys, Luc Leybaert, Johan Vereecke, Bernard Himpens, Geert Bultynck

**Affiliations:** 1 Laboratory of Molecular and Cellular Signaling, Department of Cellular and Molecular Medicine, Campus Gasthuisberg O/N-1, Faculty of Medicine, KU Leuven, Leuven, Belgium; 2 Department of Basic Medical Sciences, Physiology Group, Faculty of Medicine and Health Sciences, Ghent University, Ghent, Belgium; BioScience Project, United States of America

## Abstract

ATP-dependent paracrine signaling, mediated via the release of ATP through plasma membrane-embedded hemichannels of the connexin family, coordinates a synchronized response between neighboring cells. Connexin 43 (Cx43) hemichannels that are present in the plasma membrane need to be tightly regulated to ensure cell viability. In monolayers of bovine corneal endothelial cells (BCEC),Cx43-mediated ATP release is strongly inhibited when the cells are treated with inflammatory mediators, in particular thrombin and histamine. In this study we investigated the involvement of RhoA activation in the inhibition of hemichannel-mediated ATP release in BCEC. We found that RhoA activation occurs rapidly and transiently upon thrombin treatment of BCEC. The RhoA activity correlated with the onset of actomyosin contractility that is involved in the inhibition of Cx43 hemichannels. RhoA activation and inhibition of Cx43-hemichannel activity were both prevented by pre-treatment of the cells with C3-toxin as well as knock down of RhoA by siRNA. These findings provide evidence that RhoA activation is a key player in thrombin-induced inhibition of Cx43-hemichannel activity. This study demonstrates that RhoA GTPase activity is involved in the acute inhibition of ATP-dependent paracrine signaling, mediated by Cx43 hemichannels, in response to the inflammatory mediator thrombin. Therefore, RhoA appears to be an important molecular switch that controls Cx43 hemichannel openings and hemichannel-mediated ATP-dependent paracrine intercellular communication under (patho)physiological conditions of stress.

## Introduction

Intercellular communication can occur through direct and indirect pathways. Direct signaling is possible *via* connexin (Cx)-based gap junction channels that connect the cytoplasm of adjacent cells. An important indirect manner of intercellular communication is via paracrine signaling. Unopposed Cx-based channels present in the plasma membrane, referred to as Cx “hemichannels”, are a known conduit for the release of biomolecules, including purinergic messengers. The activity of Cx hemichannels is tightly regulated and characterized by a low open probability under physiological conditions to prevent deleterious events such as excessive loss of metabolites [1]. The latter may occur under pathological conditions like inflammation [2], [3], ischemia [4] and oxidative stress [5].

We extensively characterized gap junction- and hemichannel-mediated intercellular communication in bovine corneal endothelial cells (reviewed in [6]), a model system often used to study barrier integrity in the corneal endothelium [7]. Our previous work elucidated that intercellular communication (IC) in these cells is mainly mediated through paracrine signaling via the release of ATP through Cx hemichannels consisting of the 43-kDa isoform (Cx43) [8]–[10]. Furthermore, this ATP-driven paracrine IC is inhibited acutely and potently by the application of thrombin [6], [10]–[13]. Thrombin-induced inhibition of hemichannel responses is prevented by pre-treating the cells with either the Myosin Light Chain kinase (MLCK) inhibitor ML-7, the Rho-Associated Kinase (ROCK) inhibitor Y-27632 or the Protein Kinase C (PKC) inhibitor chelerythrine [11], [12]. These pharmacological agents have been demonstrated to prevent enhancement of MLC-phosphorylation in BCEC upon thrombin treatment [14]. By blocking myosin II-mediated cell-contraction using (–)-blebbistatin, a potent and selective inhibitor of myosin II ATPase activity, we demonstrated the necessity of the active non-muscle myosin II-dependent contractile system in the thrombin-induced inhibition of hemichannel activity in BCEC [13]. Since Rho GTPases, as molecular switches, are implicated in the regulation of actomyosin contractility through the Rho-ROCK-Myosin signaling pathway [15], [16], we wanted to investigate the possible involvement of Rho GTPase activity in the inhibitory action of thrombin on Cx43-hemichannel activity.

## Results

### C3 Prevents Inhibition of Cx43 Hemichannels by Thrombin

In a previous study, we showed that both thrombin and TRAP-6 (thrombin receptor activator peptide 6, SFLLRN) inhibit hemichannel activity through activation of the protease activated receptor 1 (PAR-1) in BCEC [11]. PAR-1 activation is known to promote cell contraction and gap formation in confluent monolayers of endothelial cells, a mechanism that involves the Rho-Rho kinase pathway [16], [17]. Since the C3-exoenzyme of *Clostridium botulinum*, potently inhibits Rho GTPase activity by ADP-ribosylation [18], we applied cell-permeable C3-transferase (referred to as “C3”) while monitoring hemichannel activity (by investigating either intercellular 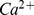-wave propagation, mechanical stimulation induced ATP release and LY uptake) in the presence and absence of thrombin.

**Figure 1:**
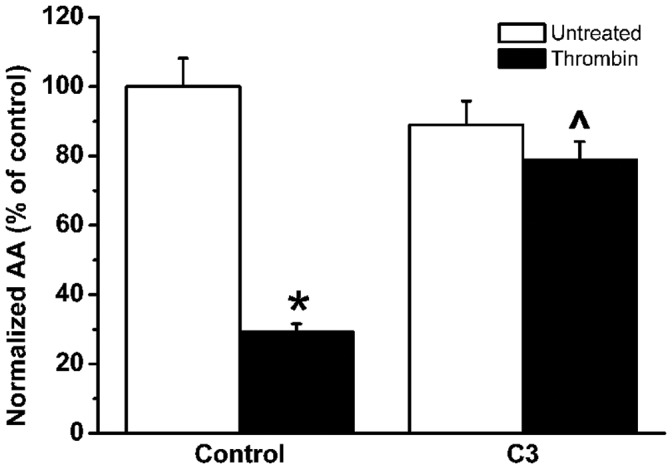
Effect of C3-pretreatment on the thrombin-induced inhibition of mechanical stimulation-induced 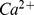-wave propagation in BCEC. Quantitative analysis of the total spreading of the mechanical stimulation-triggered 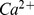 wave, presented as active area (AA) normalized to the control condition (100%) in untreated (white bars) and thrombin-treated (black bars) BCEC, either in control conditions or in C3-pretreated conditions. 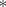 indicates that thrombin significantly (P 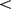 0.001) inhibits 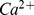-wave propagation. ^∧ ^indicates that C3 significantly (p 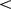 0.001) alleviates the thrombin-induced inhibition of 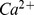-wave propagation.

In intercellular 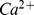-wave propagation experiments with BCEC, the mechanically stimulated cell shows a transient 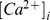 rise, which originates at the point of stimulation and spreads out to the neighboring cells in a wave-like manner. 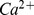-transients were observed up to approximately four to six cell layers away from the mechanically stimulated cell. Thrombin-treated BCEC also show intercellular propagation of the 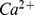 wave, but the spread of the wave is strongly reduced to about two to four cell layers. Figure 1 shows the quantitative analysis of spreading of the 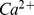 wave, presented as “Normalized Active Area (AA)” (Fig. 1). Thrombin-treatment reduced the AA by 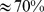 (19.3 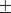 1.5 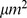 (N = 5, n = 48) versus 66.3 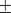 5.4 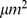 under control conditions (N = 5, n = 48)).

No significant difference was found in AA between control and C3-pretreated BCEC in the absence of thrombin (comparison of black bars in Fig. 1). C3-pretreatment of BCEC significantly suppressed the effect of thrombin on the propagation of the 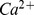-wave (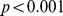; N = 5, n = 48; comparison grey bars, Fig. 1). Taken together, these findings demonstrate that Rho GTPase activity is involved in the thrombin-induced inhibition of 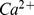-wave propagation.

**Figure 2:**
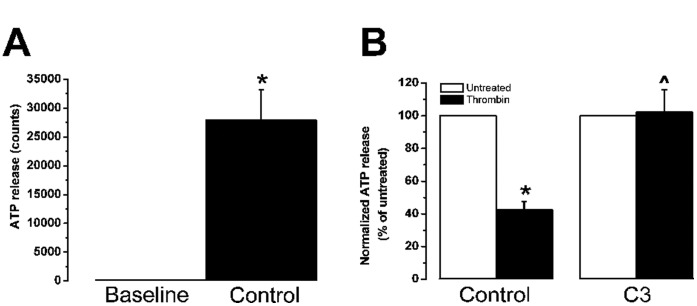
Effect of C3-pretreatment on the thrombin-inhibited release of ATP in BCEC. Quantitative measurement of mechanical stimulus-induced ATP release using the bioluminescence luciferin-luciferase assay. (A) Mean number of light counts under baseline (no stimulation) and after mechanical stimulation (control condition) were quantified (N = 5, n = 11). (B) Normalized ATP release after mechanical stimulation of untreated (white bars) and thrombin-treated (black bars) BCEC, either in control conditions (N = 5, n = 11) or in C3-toxin-pretreated conditions (N = 5, n = 9), is shown. 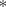 indicates that thrombin significantly (p 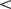 0.001) inhibits 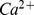-wave propagation. ^∧ ^indicates that C3-toxin significantly (p 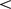 0.001) alleviates the thrombin-induced inhibition of 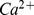-wave propagation.

**Figure 3:**
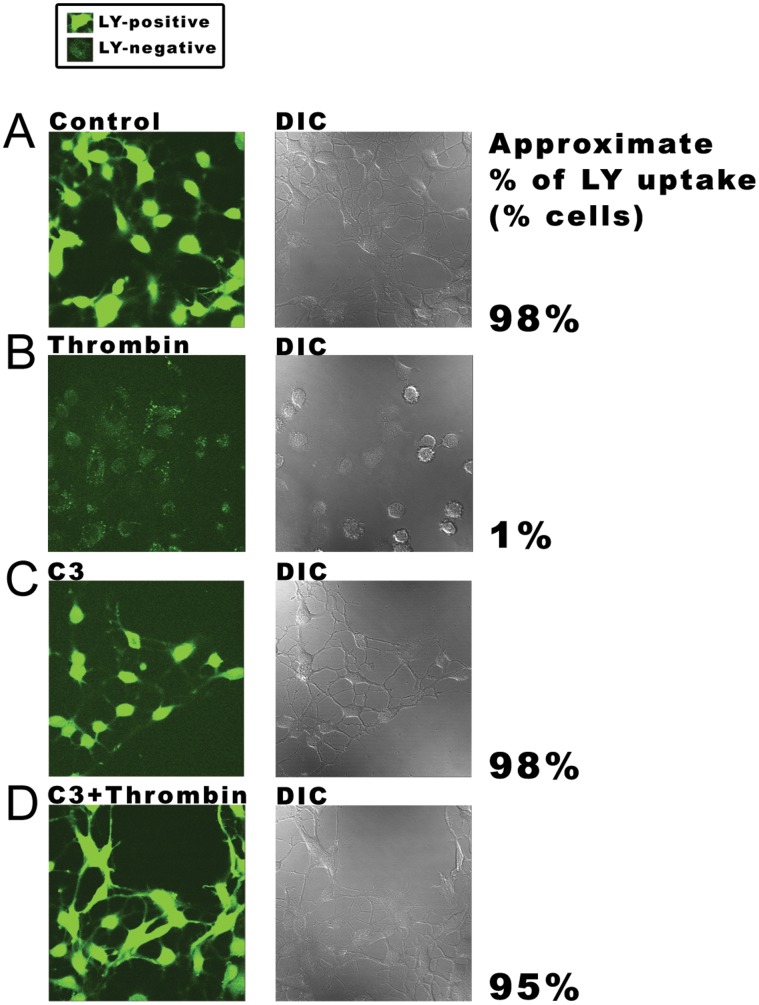
LY-uptake after C3-pretreatment in the absence and presence of thrombin. Non-confluent grown BCEC were exposed to the hydrophilic Lucifer Yellow dye (2.5% for 5 min) in a PBS-buffer containing 2 mM EGTA. Left panels represent the fluorescence signal of LY while the cells are visualized by differential interference contrast (DIC) microscopy (right panels). (A) Uptake of LY in control condition (N = 4, n = 20). (B) LY-uptake is blocked in BCEC treated with thrombin (2 U/ml) for 5 min (N = 4, n = 20). (C) LY-uptake after pretreatment with C3 (2 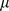
g/ml) for 3 hours (N = 4, n = 20). (D) C3 prevents significant LY-uptake after thrombin-treatment (N = 4, n = 20). The approximate percentage of cells that accumulated the dye was determined for each condition by manually counting the number of LY-positive and LY-negative cells (n = 200 cells). Cells were considered LY-positive or -negative as indicated in the box.

Since the hemichannel-mediated responses of BCEC in 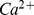-wave propagation experiments are largely dependent on ATP release [8], [9], [11], [13] via Cx43 hemichannels [10], and because thrombin was shown previously to reduce ATP release upon mechanical stimulation [10]–[13], the effect of C3-pretreatment on ATP release upon mechanical stimulation was examined. Thrombin reduced ATP release upon mechanical stimulation by 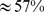 (N = 5, n = 11, Fig 2), while thrombin did not reduce ATP release after C3-pretreatment (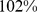 ATP release versus 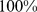 ATP release in control conditions (N = 5, n = 9), Fig. 2). These results provide evidence that thrombin-enhanced Rho GTPase activity is involved in the thrombin-induced inhibition of Cx43-hemichannel mediated ATP release. We also investigated the effect of C3 on the opening of Cx hemichannels triggered by exposure of BCEC to a 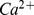-free solution containing EGTA, by visualizing the uptake of the hydrophilic dye Lucifer Yellow (LY). As shown previously [11], [12], BCEC take up LY under control conditions and thrombin potently inhibits this LY uptake (Fig. 3A and B). However, when cells were pretreated with C3, thrombin did not inhibit the uptake of LY (Fig. 3D). Since C3 can prevent the inhibitory effect of thrombin on Cx-hemichannel activity, these results demonstrate the involvement of a Rho GTPase in the signaling cascade triggered by thrombin.

### C3 Prevents RhoA Activation

**Figure 4:**
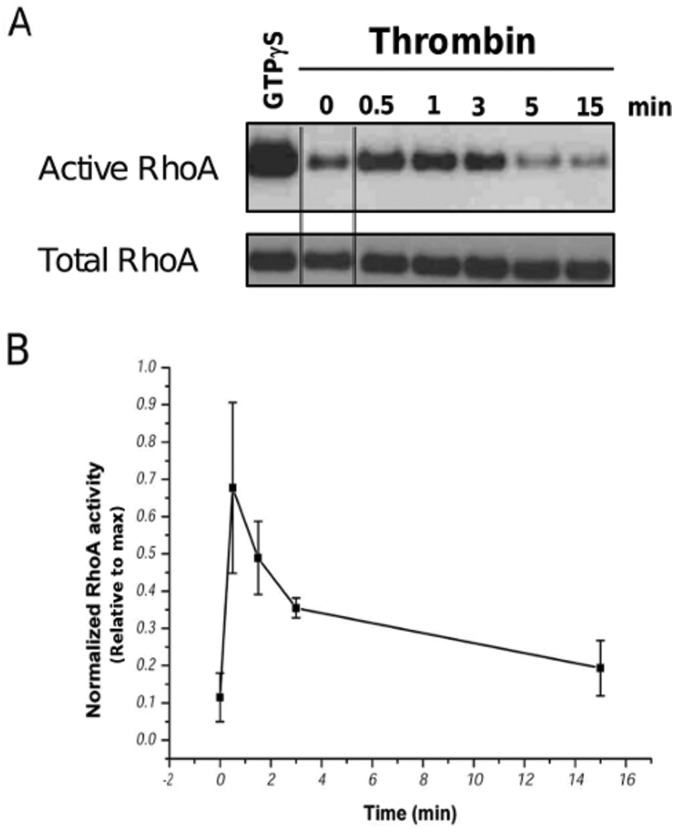
Thrombin-stimulated RhoA activity in BCEC lysates as a function of time. Transient RhoA activation in BCEC after treatment with thrombin (2 U/ml). Freshly prepapred lysates from BCEC before thrombin exposure (0 min) and after 0.5 min, 1 min, 3 min, 5 min and 15 min after thrombin exposure were assessed. Active RhoA was isolated using GST-Rhotekin-RBD beads following the manufacturer’s protocol. In vitro GTP
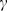
S-loaded RhoA was used as a positive control (square inset). Both the active RhoA levels as well as the total RhoA levels were analyzed by immunoblotting using anti-RhoA. The double lines indicate samples were taken from a different part from the same blot and exposure. (B) Quantitative analysis of 3 independent experiments, in which the activated RhoA signals were normalized to the signal of the maximal activatable RhoA obtained using GTP
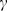
S treatment. Data represent mean 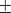 S.E.M.

**Figure 5:**
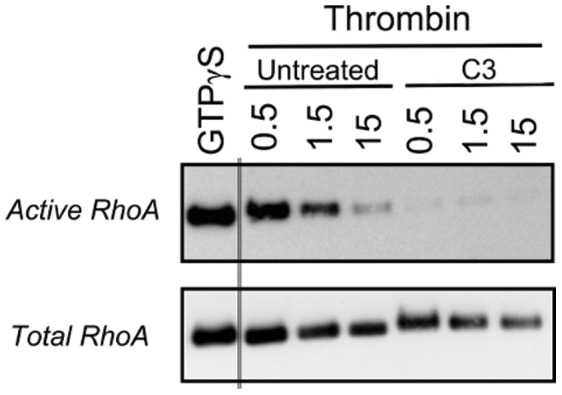
Thrombin-stimulated RhoA activity in BCEC after C3-pretreatment. Western-blot analysis of RhoA activity after BCEC exposure to thrombin (0.5, 1.5 and 15 min) obtained from lysates of untreated BCEC and BCEC pretreated for 3 hours with C3 at 37°C. Active RhoA fraction was isolated from freshly prepared BCEC lysates using GST-Rhotekin-RBD beads following the manufacturer’s protocol (upper blot). In vitro GTP
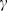
S-loaded RhoA was used as a positive control. The total RhoA levels were determined in all lysates (lower blot).

Thrombin induces rapidly the activation of RhoA (a Rho GTPase family member) in endothelial cells [19]. Since RhoA activation promotes cell contractility by the actin-myosin II system via the ability of RhoA to regulate the MLC-phosphorylation status (Rho/ROCK axis), it is plausible that RhoA is an important Rho GTPase protein involved in the thrombin-induced inhibition of Cx43-hemichannel activity. Therefore, we measured the time course of RhoA activation upon stimulation of BCEC with thrombin for various periods. GTP-bound Rho GTPase proteins were pulled down from lysates of BCEC using the Rho-binding domain of human Rhotekin fused to GST (Rhotekin-RBD beads) and Western blot analysis was performed to monitor active (GTP-bound) and total RhoA levels in time. Active RhoA levels rapidly increased (within 30 s) after thrombin application. After 5 min, active RhoA levels already declined (Fig. 4). When thrombin-treated BCEC that were pretreated with C3, were analyzed for their levels of active RhoA using the pull down method, only low levels of active RhoA were detected (Fig. 5), when compared to maximal RhoA-activation levels induced by loading of RhoA with a non-hydrolyzable GTP-analogue (GTP
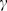
S).

### RhoA Knockdown Overcomes Thrombin-induced Inhibition of Cx43-hemichannel-driven Intercellular Communication

**Figure 6:**
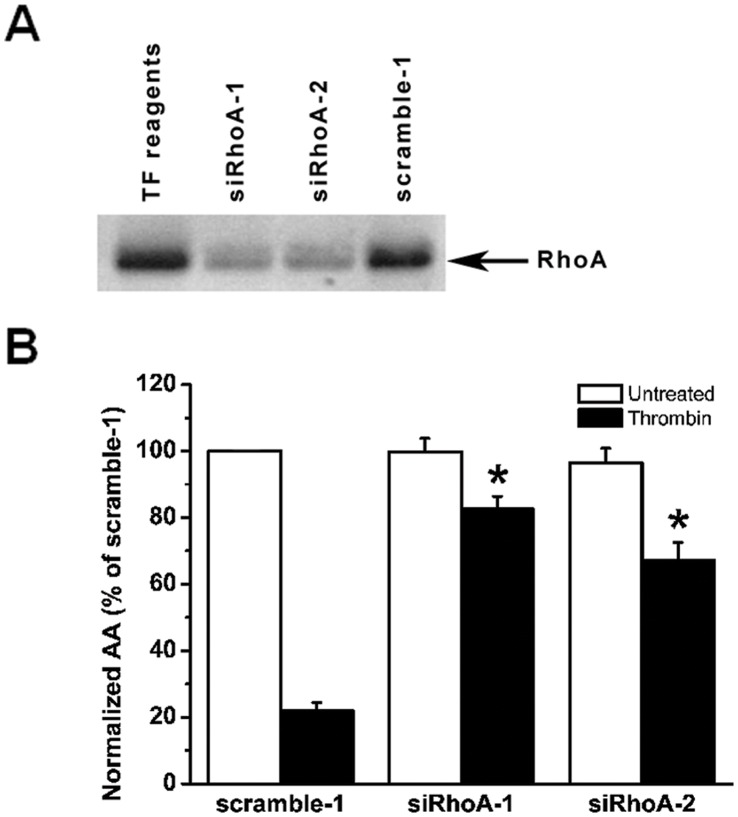
Effect of RhoA knockdown on thrombin-induced inhibition of mechanical stimulus-induced 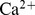-wave propagation in BCEC. (A) Representative Western-blot analysis showing total RhoA-protein levels in total cell lysates (20 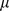
g/lane) of BCEC treated with siRNA-1 against RhoA (siRhoA-1), siRNA-2 against RhoA (siRhoA-2) and siRNA representing a scrambled version of siRNA-1 against RhoA (scramble-1). (B) Quantitative analysis of the total spreading of the mechanical stimulation-triggered 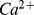-wave in siRNA treated cells, presented as active area (AA) normalized to the untreated (white bar) scramble-1 condition (100%). Comparison of the thrombin-treated conditions (black bars) indicates that both siRhoA-1 and siRhoA-2 independently and significantly alleviated the thrombin-induced inhibition of the AA of the intercellular 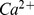-wave propagation in comparison to scramble-1-treated samples (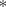, p 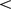 0.001) (N = 3, n = 30).

To examine the involvement of RhoA in thrombin-induced inhibition of Cx hemichannel activity, we performed 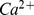-wave propagation experiments on BCEC approximately 24 hours after they were transfected with RhoA-targetting siRNA. We previously have shown that under these conditions more than 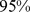 of the cells have taken up the siRNA duplexes [10]. In addition, to minimize off-target effects, we examined independently the effects of two siRNA-duplexes that target different regions of the RhoA mRNA. Efficiency of RhoA knockdown by both siRNA-duplexes was verified by Western-blotting analysis (Fig. 6A), which show a more than 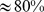 reduction in RhoA-expression levels in comparison to cells transfected with the scrambled siRNA control version. Both siRhoA-duplexes independently overcome the thrombin-induced inhibition of Cx hemichannel responses, while hemichannel activity is potently inhibited by thrombin in BCEC transfected with scrambled siRNA-duplex (Fig. 6B). These results demonstrate that RhoA GTPase activity is involved in the thrombin-induced inhibition of Cx hemichannels in BCEC.

**Figure 7:**
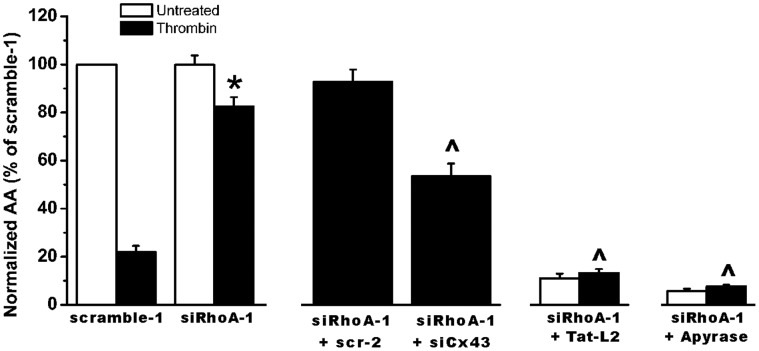
Effect of Cx43 knockdown, TAT-Cx43L2 and apyrase treatment on the siRhoA-opposed thrombin-induced inhibition of 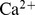-wave propagation in BCEC. Quantitative analysis of the total spreading of the mechanical stimulation-triggered 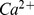-wave in thrombin-treated cells as active area (AA) normalized to the untreated (no thrombin, white bar) scramble-1 condition (100%). Knockdown of RhoA by siRhoA-1 significantly prevented the thrombin-induced inhibition of 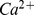-wave propagation (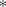, p 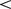 0.001). Simultaneous knock down of Cx43 (siCx43) and RhoA (siRhoA-1) significantly reduced the normalized AA when compared to siRhoA-1 only or siRhoA-1 plus scr-2. Scr-2 represents treatment with a scrambled version of siCx43 siRNA. Experiments with siRNA-treated cells in the presence of either TAT-Cx43L2 or apyrase show that both TAT-Cx43L2 and apyrase potently reduced the normalized AA in BCEC upon RhoA knockdown in the presence (^∧^, p 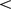 0.001, i.e. comparison of black bars) and absence of thrombin (N = 3, n = 30).

To demonstrate the involvement of Cx43-made channels in the downstream cascade of activated RhoA, we performed 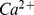-wave propagation experiments with thrombin in conditions where RhoA and Cx43 were simultaneously knocked down. siRhoA-1 does not completely rescue the active area in the presence of thrombin when Cx43 is knocked down. The results show that Cx43 is involved in the RhoA-signaling cascade because the siRhoA-mediated alleviation of thrombin-induced 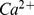-wave propagation was significantly reduced by siCx43, and the reduction is significantly larger than the active area of the condition with thrombin-stimulated cells that were transfected with control siRNA (scramble-1) (Fig. 7).

Since the knock-down approach with siRNA against Cx43 affects both Cx43-hemichannel- and - gap junction-mediated signaling, we performed additional experiments to investigate an eventual contribution of the gap junctional pathway, although it was previously demonstrated that the gap junctional pathway only has a limited contribution to intercellular 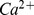-waves propagation in BCEC ([8], [10], [11], [20]. Therefore, we investigated the effect of thrombin in conditions of RhoA-knockdown where intercellular 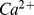-wave propagation mediated by Cx43 hemichannels was inhibited while gap junctional communication was unaffected. In a first set of experiments, we applied the TAT-Cx43L2 peptide. This Cx43-mimetic peptide, derived from a sequence located in the intracellular loop of Cx43, has been validated to selectively block Cx43-hemichannels while maintaining gap junctional coupling [10], [21] (Fig. 7). TAT-Cx43L2 potently reduced the active area of the 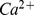-wave in siRhoA-1 treated cells. No significant further reduction was detected when also thrombin was applied to these cells, which confirms the involvement of Cx43-hemichannels in the signaling cascade.

In another set of experiments, we investigated the effect of thrombin after knock-down of RhoA when ATP-dependent paracellular communication was blocked by extracellular apyrases (Fig. 7). A mixture of ectonucleotidases apyrase VI (5 U/ml) and VII (5 U/ml) was used, which is known to potently block all ATP/ADP dependent purinergic signaling and to strongly reduce propagation of the 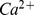 wave in monolayers of BCEC [9]–[11]. Blocking the Cx hemichannel-mediated ATP-dependent paracrine pathway after RhoA knock-down by either TAT-Cx43L2 or apyrases resulted in an active area that was strongly reduced without further reduction of the active area upon thrombin treatment (Fig. 7). This demonstrates that the effect of RhoA knock-down is virtually exclusively mediated via the pathway that involves ATP-dependent paracrine intercellular communication through Cx43 hemichannels. The results of these experiments indicate that knockdown of RhoA upon blocking the purinergic signaling in a Cx43-dependent and -independent manner while maintaining gap junctional coupling does not significantly oppose the thrombin-induced inhibition of 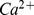-wave propagation. This suggests that possible contribution of activated RhoA in inhibiting Cx43 gap junctional coupling in thrombin-treated BCEC is negligible (Fig. 7). These findings, taken together with our previous finding that thrombin inhibits Cx43-hemichannel responses in BCEC [10] by enhanced contractility [13], provide evidence that RhoA is a key component in contractility-induced-inhibition of Cx43-hemichannel responses.

## Discussion

**Figure 8:**
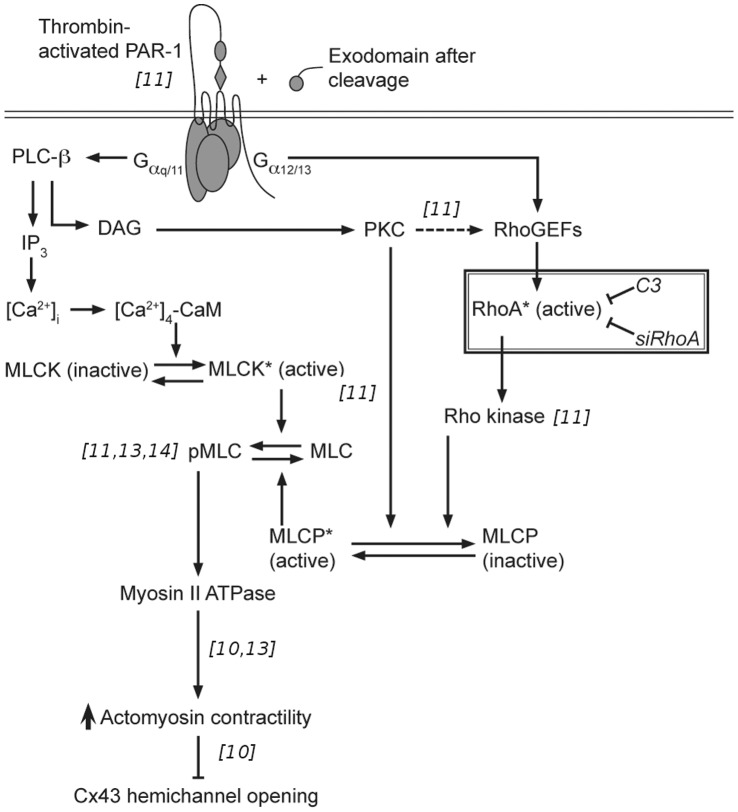
Signal-transduction cascade of thrombin-induced inhibition of Cx43 hemichannel opening in BCEC. Thrombin-mediated activation of the cell-surface-expressed protease-activated receptor-1 (PAR-1) affects the MLC phosphorylation-state through multiple intracellular signaling pathways. The effector response of PAR-1 depends on the isoform of the G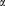 subunit (i.e. G
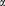
q/11 or G
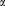
12/13) that is coupled to this G-protein-coupled receptor. These pathways include PKC-dependent signaling, IP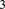-dependent 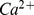 rise and activation of the 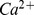-sensitization pathway via RhoA-ROCK signaling. Enhanced MLC-phosphorylation leads to increased actomyosin II dependent contractility and subsequently inhibition of Cx43 hemichannel opening. The findings in this study described that RhoA activation is essential to cause contractility-induced inhibition of the Cx43 hemichannel. References, included in the figure within italic brackets, indicate in which study we provide the supporting experimental data.

The most important finding of this study is that RhoA is an essential component of the signaling cascade that controls inhibition of Cx43-hemichannel activity through contractility and the hemichannel-mediated ATP-dependent paracrine intercellular communication. We show that RhoA is activated upon exposure of BCEC to thrombin and that chemical inhibition of RhoA activity with C3 and genetic inhibition of RhoA expression with siRNA alleviates the inhibition of Cx43 hemichannels by increased contractility in BCEC induced by thrombin exposure. These findings are important, since the Rho-ROCK signaling axis controls the thrombin-induced activation of contractility by non-muscle myosin II. Our findings indicate that thrombin activates RhoA in BCEC cells and that C3-treatment prevents the inhibitory effect of thrombin on intercellular communication as assayed by 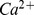-wave propagation, ATP release and LY-uptake. The C3-transferase of *Clostridium botulinum* is known to covalently modify RhoA, RhoB and RhoC GTPases by mono-ADP-ribosylation. This event inactivates these Rho GTPases by preventing nucleotide exchange [18]. Pre-treatment of BCEC for 3 hours caused robust ADP-ribosylation of RhoA, which is characterized by an increase in molecular weight and subsequently a shift in migration-pattern that becomes clear after RhoA-immunoblotting. Since we demonstrated that pre-treatment of the cells with the C3-exoenzyme prevents the thrombin-induced inhibition of Cx43-hemichannel responses without significantly affecting intercellular 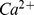-wave propagation and mechanically-induced ATP-release from BCEC in the absence of thrombin, Rho GTPases are clearly involved in the intracellular signaling cascade that brings about contractility-induced inhibition of Cx43-hemichannel activity (Figs. 1 and 2). These results, which are in line with dye uptake experiments performed with BCEC that were pretreated with C3 (Fig. 3), indicate that at least one Rho GTPase is involved in the thrombin-induced inhibition of Cx43-channel activity in BCEC. By performing reverse-transcriptase PCR experiments we detected the presence of RhoA and RhoB in BCEC. These results were confirmed by Western blot analysis (data not shown). The presence of the Rho GTPase effector ROCK-II ( = ROK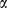) but not ROCK-I ( = ROK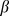  = p160-ROCK) was detected in BCEC at the mRNA-level (data not shown). Since it is known that the ROCK-II-inhibitor Y-27632 could prevent the inhibitory effects of thrombin on Cx43-mediated gap junction - and hemichannel activity [11], it is very likely that ROCK-II is a major effector kinase in the signaling cascade that causes actomyosin contractility in BCEC. We propose that RhoA has an essential role in the studied signaling cascade (presented in Fig. 8) because we found that *i)* RhoA-GTPase activity is rapidly and transiently increased within 1 minute after thrombin exposure of BCEC, *ii)* C3-treatment, which prevented RhoA activation, alleviated thrombin-induced inhibition of Cx43-hemichannel responses, and *iii)* suppressing RhoA expression using siRNA completely alleviated thrombin-induced inhibition of Cx43-channel responses. Therefore, we propose that RhoA GTPases act as a molecular switch that controls the onset of thrombin-induced actomyosin contractility causing the potent inhibition of Cx43-hemichannel activity and subsequent ATP release. Furthermore, while several studies described that thrombin-induced signaling through RhoA leads to ATP release [22]–[28], our result indicate that RhoA activation in response to thrombin can prevent ATP release. The apparent discrepancy between both observations may be due to a difference in the molecular signaling intermediates (e.g. ROCK independent) [22], [23] and release mechanisms (e.g. exocytosis) [26] that are responsible for ATP release. While Cx43 hemichannels are inhibited by thrombin-induced signaling by activation of the actomyosin cytoskeleton, other Cx or Panx isoforms may not or differentially be influenced through this pathway [27]. In this respect, a recent report from thrombin-treated human umbilical vein endothelial cells showed that thrombin induced ATP release was mediated by Panx1 channels, but not by Cx43 hemichannels [28].

While our findings suggest that possible contribution of activated RhoA in inhibiting Cx43 gap junctional coupling in thrombin-treated BCEC is likely negligible or at least difficult to detect, we do not exclude that in other cell-systems activated RhoA might be able to potently regulate gap junctional coupling. Moreover, data from various authors underpin the concept that Rho GTPases and cytoskeletal reorganization are often involved in mechanisms that influence gap junctional - or hemichannel-mediated communication [22]–[24], [27]–[29].

Recently, we have documented possible mechanisms and relevance by which enhanced actomyosin contractility could negatively regulate Cx43 hemichannel responses [30]. Experiments with C-terminal peptides of Cx43 (TAT-Cx43CT10) in cells that overexpress either wild-type Cx43 or C-terminal truncated Cx43 identified the C-terminal tail of Cx43-hemichannels as a potential link to the contractile system and indicate that contractility disrupts Cx43 loop/tail interactions that are essential for ensuring proper Cx43 hemichannel responses [10]. While the exact molecular mechanism has not yet been resolved, the C-terminal tail of Cx43 channels in the hemichannel-conformation could sense enhanced actomyosin contractility in either a direct or indirect manner. One or more Cx43-binding proteins that interact with the C-terminal tail could provide the link with the actin cytoskeleton. The C-terminal tail might also be indirectly coupled to the actin cytoskeleton by interacting with another membrane-associated protein that senses the actomyosin contractility. Furthermore, it remains to be investigated whether thrombin causes a redistribution of Cx43-channels within the plasma membrane or increases internalization by an actomyosin-dependent mechanism. Anderson et al. described that inhibition of ROCK by Y-27623 increased the number of Cx43 gap junctions and improved gap junctional intercellular communication between rabbit corneal epithelial cells [31]. In addition, GPCR-activation induced GJ-channel closure within 1–2 minutes [32], while GJ-internalization can occur within 10 to 20 minutes [33]. PAR-1 activation by thrombin rapidly inhibited GJ-channels, which coincided with the internalization of gap junctions in porcine pulmonary artery endothelial cells. The mechanism appears to involve the endocytosis-associated vesicle-coat protein clathrin and the cytoskeleton-associated scaffold ZO-1 [33].

Our results obtained by experiments with pharmacological inhibitors (Y-27623 and blebbistatin) [10], [11], [13], which act downstream of RhoA, indicate that contractility mediated by the actomyosin II system is essential for the thrombin-induced inhibition of Cx43 hemichannels. These data largely exclude that a contractility-independent effect of RhoA causes the inhibition of Cx43-hemichannels by thrombin.

In conclusion, our current findings demonstrate that besides the previously identified Rho-associated kinase [11], MLC phosphorylation [11], [13] and actomyosin II-mediated contractility [13], RhoA activation is an essential step in the cascade underlying thrombin-induced inhibition of Cx43-hemichannel-mediated ATP release through activation of actomyosin contractile system [6], [10], [30] (Fig 8).

## Materials and Methods

### Cell Culture

Primary cultures of BCEC were established as previously described [8], [11], [13]. First and second passage cells were seeded into two-chambered glass slides (155380; Nunc, Roskilde, Denmark) at a density of 165,000 cells per chamber (4.2 cm^2^) and were grown for three to four days to reach confluence.

### Chemicals

Fluo-4 AM (F14217), Dulbecco’s PBS (14190-091), Dulbecco’s modified Eagle’s medium (DMEM, 11960-044), L-glutamine (Glutamax, 35050-038), antibiotic antimycotic mixture (15240-096), EBSS (Earle’s balanced salt solution, 14155-048), and trypsin (25300-054) were obtained from Invitrogen-Gibco. Fetal bovine serum (F-7524), a luciferin-luciferase bioluminescence assay kit (FL-AAM), ATP, thrombin (T-4648), apyrase VI (A6410), apyrase VII (A6535) and LY (L-0259) were obtained from Sigma-Aldrich (Deisenhofen, Germany). The cell-permeable Rho inhibitor exoenzyme C3-transferase (CT04), referred to as “C3”, was obtained from Cytoskeleton, Inc (Denver, CO, USA).

### TAT-Cx43L2 Peptide

TAT-Cx43L2 peptide (85% pure) was obtained from Lifetein (South Plainfield, NJ, USA). TAT-Cx43L2 (YGRKKRRQRRRDGANVDMHLKQIEIKKFKYGIEEHGK) was used at 100 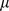
M and incubated with the cells for 30 min at 37°C as described previously [10].

### Imaging of 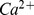-wave Propagation

The protocol for point mechanical stimulation of a single cell has been described in our previous studies [8], [11], [13]. The mechanical stimulation consisted of an acute deformation of a cell by briefly touching less than 1% of its cell membrane with a glass micropipette (tip diameter 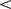 1 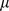
m) coupled to a piezoelectric crystal (Piezo device P-280, Amplifier-E463; PI Polytech, Karlsruhe, Germany). The 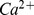-wave propagation was assayed by imaging 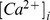. Therefore, BCEC were loaded with the 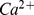-sensitive fluorescent dye Fluo-4-AM for 30 min at 37°C on a rocking shaker (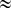 10 rpm). BCEC were pre-incubated in the presence (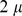
g/ml) or absence of the cell-permeable Rho inhibitor (C3) for three hours at 37°C and 5% 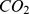. Pre-incubation, Fluo-4 loading and experiments were performed in PBS containing 0.9 mM 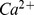 and 25 mM glucose. After Fluo-4 loading, BCEC were exposed to thrombin (2 U/ml) and 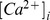 levels were allowed to recover to basal levels before performing the 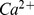-wave propagation experiment. Fluo-4 was excited at 488 nm, and its fluorescence emission was collected at 530 nm. Spatial changes in 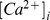 following point mechanical stimulation were measured with the confocal microscope (LSM510) using a 40X objective (1.3 NA). Intercellular propagation of the 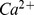 wave was characterized by the total surface area of responsive cells (active area, AA) with normalized fluorescence 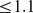 using imaging software (LSM Image 4.2, Zeiss).

### Lucifer Yellow Uptake Assay

BCEC were treated with cell-permeable C3 (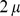
g/ml) for 3 hours under same physiological conditions as used for 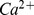-wave propagation. Then, cells were incubated in LY-solution (PBS solution containing 2 mM EGTA and 2.5% LY) for 5 min. In experiments with thrombin, cells were exposed to thrombin for one minute before exposure to LY. Dye uptake was recorded using the confocal microscope (LSM510; Carl Zeiss) by excitation at 488 nm with emission recorded at 530 nm as described previously [11]–[13].

### Measurement of ATP Release

ATP release induced by mechanical stimulation (performed on the confocal microscope for visualization of the stimulus), was measured using the luciferin-luciferase bioluminescence technique as previously described [8], [11], [12]. Aliquots of 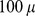
l were taken from the 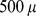
l bathing solution covering the cells, and transferred to a custom-built photo-counting setup [13] for quantification of ATP-amount. BCEC were treated with C3 for 3 hours under same physiological conditions as used for 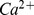-wave propagation.

### RhoA-activity Assay

Activation of Rho GTPase was examined by GST pull-down of GTP-bound Rho proteins by using the Rho-binding domain (RBD) of Rhothekin protein (an effector of RhoA) expressed as a GST-fusion protein and coupled to agarose beads (RT02, Cytoskeleton, Inc.) to capture the active (GTP-bound) Rho proteins in BCEC-lysates. Pull down samples were subjected to SDS-PAGE and Western-blotting analysis. Active and total RhoA levels were visualized after immunoblotting with a primary rabbit monoclonal anti-RhoA antibody (clone 67B9, 2117, Cell Signaling Technology, Beverly, MA, USA).

### Knock-down of RhoA and Cx43 by siRNA

Screening for potent siRNA-oligonucleotides, with exclusion of off-target candidates to selectively knock-down RhoA in BCEC, was performed using the RNAi batch selector [34] on a FreeBSD system. Two siRNA-duplexes that target different regions of RhoA mRNA (sense siRhoA-1∶5′CUAUGUGGCAGAUAUUGAdTdT; sense siRhoA-2∶5′UUAGGCUGUAACUACUUUAdTdT) were selected for experiments and negative control was designed (sense ScramRhoA-1∶5′CUUAUAGGAUAGUGUAACAdTdT). Annealed siRNA was purchased from Eurogentec (Liege, Belgium) and BCEC were transfected with siRNA duplexes (80 nM final) for 6 hours in Optimem medium (Invitrogen) using HiPerfect transfection reagent (Qiagen, Venlo, The Netherlands). Knockdown was examined by Western blot analysis using a rabbit monoclonal (clone 67B9) anti-RhoA antibody (Cell Signaling Technology). BCEC were transfected with si1Cx43 duplex or its negative control (scr-2) at 80 nM final, as previously described and validated (sense siCx43-1∶5′GAAGGAGGAGGAACUCAAAdTdT, sense scr-2∶5′GGUAAACGGAACGAGAAGAdTdT) [10].

### Data Analysis

One-way ANOVA was used to compare the mean values for different treatments while unpaired tests were used to compare results of experiments with a single treatment and a single control. A p value of 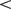 0.05 is considered statistically significant. Histograms are expressed as mean 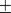 standard error of the mean (SEM). “N” indicates the number of independent experiments, while “n” represents the total number of mechanically stimulated cells.

## References

[1] Sáez JC, Schalper KA, Retamal MA, Orellana JA, Shoji KF, et al (2010) Cell membrane permeabilization via connexin hemichannels in living and dying cells. Exp Cell Res 316: 2377–2389. 20595004 10.1016/j.yexcr.2010.05.026

[2] Retamal MA, Froger N, Palacios-Prado N, Ezan P, Sáez PJ, et al (2007) Cx43 hemichannels and gap junction channels in astrocytes are regulated oppositely by proin–ammatory cytokines released from activated microglia. J Neurosci 27: 13781–13792. 18077690 10.1523/JNEUROSCI.2042-07.2007 PMC6673621

[3] Orellana JA, Sáez PJ, Shoji KF, Schalper KA, Palacios-Prado N, et al (2009) Modulation of brain hemichannels and gap junction channels by pro-inflammatory agents and their possible role in neurodegeneration. Antioxid Redox Signal 11: 369–399. 18816186 10.1089/ars.2008.2130 PMC2713807

[4] Retamal MA, Schalper KA, Shoji KF, Orellana JA, Bennett MVL, et al (2007) Possible involvement of different connexin 43 domains in plasma membrane permeabilization induced by ischemiareperfusion. J Membr Biol 218: 49–63. 17705051 10.1007/s00232-007-9043-y

[5] Retamal MA, Schalper KA, Shoji KF, Bennett MVL, Sáez JC (2007) Opening of connexin 43 hemichannels is increased by lowering intracellular redox potential. Proc Natl Acad Sci U S A 104: 8322–8327. 17494739 10.1073/pnas.0702456104 PMC1895948

[6] Ponsaerts R, D’hondt C, Gomes P, Bultynck G, Srinivas SP, et al. (2010) Extracellular ATP and adenosine as regulators of endothelial cell function: Implications for health and disease, Springer Science, volume 1, chapter 10: ATP release via connexin hemichannels controls intercellular propagation of Ca2+ waves in corneal endothelial cells. 1st edition, 161–203.

[7] Srinivas SP (2010) Dynamic regulation of barrier integrity of the corneal endothelium. Optom Vis Sci 87: E239–E254. 20142793 10.1097/OPX.0b013e3181d39464 PMC2868144

[8] Gomes P, Srinivas SP, Van Driessche W, Vereecke J, Himpens B (2005) ATP release through connexin hemichannels in corneal endothelial cells. Invest Ophthalmol Vis Sci 46: 1208–1218. 15790881 10.1167/iovs.04-1181

[9] Gomes P, Srinivas SP, Vereecke J, Himpens B (2005) ATP-dependent paracrine intercellular communication in cultured bovine corneal endothelial cells. Invest Ophthalmol Vis Sci 46: 104–113. 15623761 10.1167/iovs.04-0846

[10] Ponsaerts R, De Vuyst E, Retamal M, D’hondt C, Vermeire D, et al (2010) Intramolecular loop/tail interactions are essential for connexin 43-hemichannel activity. FASEB J 24: 4378–4395. 20634352 10.1096/fj.09-153007

[11] D’hondt C, Ponsaerts R, Srinivas SP, Vereecke J, Himpens B (2007) Thrombin inhibits intercellular calcium wave propagation in corneal endothelial cells by modulation of hemichannels and gap junctions. Invest Ophthalmol Vis Sci 48: 120–133. 17197525 10.1167/iovs.06-0770

[12] D’hondt C, Srinivas SP, Vereecke J, Himpens B (2007) Adenosine opposes thrombin-induced inhibition of intercellular calcium wave in corneal endothelial cells. Invest Ophthalmol Vis Sci 48: 1518–1527. 17389480 10.1167/iovs.06-1062

[13] Ponsaerts R, D’hondt C, Bultynck G, Srinivas SP, Vereecke J, et al (2008) The myosin II ATPase inhibitor blebbistatin prevents thrombin-induced inhibition of intercellular calcium wave propagation in corneal endothelial cells. Invest Ophthalmol Vis Sci 49: 4816–4827. 18614806 10.1167/iovs.07-1533

[14] Satpathy M, Gallagher P, Lizotte-Waniewski M, Srinivas SP (2004) Thrombin-induced phosphorylation of the regulatory light chain of myosin II in cultured bovine corneal endothelial cells. Exp Eye Res 79: 477–486. 15381032 10.1016/j.exer.2004.06.018

[15] Somlyo AP, Somlyo AV (2003) Ca^2+^ sensitivity of smooth muscle and non-muscle myosin II: modulated by G-proteins, kinases, and myosin phosphatase. Physiol Rev 83: 1325–1358. 14506307 10.1152/physrev.00023.2003

[16] Birukova AA, Smurova K, Birukov KG, Kaibuchi K, Garcia JGN, et al (2004) Role of Rho GTPases in thrombin-induced lung vascular endothelial cells barrier dysfunction. Microvasc Res 67: 64–77. 14709404 10.1016/j.mvr.2003.09.007

[17] Vouret-Craviari V, Grall D, Obberghen-Schilling EV (2003) Modulation of rho GTPase activity in endothelial cells by selective proteinase-activated receptor (PAR) agonists. J Thromb Haemost 1: 1103–1111. 12871383 10.1046/j.1538-7836.2003.00238.x

[18] Aktories K, Wilde C, Vogelsgesang M (2004) Rho-modifying C3-like ADP-ribosyltransferases. Rev Physiol Biochem Pharmacol 152: 1–22. 15372308 10.1007/s10254-004-0034-4

[19] van Nieuw Amerongen GP, van Delft S, Vermeer MA, Collard JG, van Hinsbergh VW (2000) Activation of RhoA by thrombin in endothelial hyperpermeability: role of Rho kinase and protein tyrosine kinases. Circ Res 87: 335–340. 10948069 10.1161/01.res.87.4.335

[20] Gomes P, Srinivas SP, Vereecke J, Himpens B (2006) Gap junctional intercellular communication in bovine corneal endothelial cells. Exp Eye Res 83: 1225–1237. 16938292 10.1016/j.exer.2006.06.012

[21] Stehberg J, Moraga-Amaro R, Salazar C, Becerra A, Echeverría C, et al (2012) Release of gliotransmitters through astroglial connexin 43 hemichannels is necessary for fear memory consolidation in the basolateral amygdala. FASEB J Epub. 10.1096/fj.11-198416 22665389

[22] Kreda SM, Seminario-Vidal L, van Heusden C, Lazarowski ER (2008) Thrombin-promoted release of UDP-glucose from human astrocytoma cells. Br J Pharmacol 153: 1528–1537. 18204471 10.1038/sj.bjp.0707692 PMC2437904

[23] Blum AE, Joseph SM, Przybylski RJ, Dubyak GR (2008) Rho-family GTPases modulate ca^2+^- dependent ATP release from astrocytes. Am J Physiol Cell Physiol 295: C231–C241. 18495810 10.1152/ajpcell.00175.2008 PMC2493544

[24] Seminario-Vidal L, Kreda S, Jones L, O’Neal W, Trejo J, et al (2009) Thrombin promotes release of ATP from lung epithelial cells through coordinated activation of Rho- and Ca^2+^-dependent signaling pathways. J Biol Chem 284: 20638–20648. 19439413 10.1074/jbc.M109.004762 PMC2742828

[25] Blum AE, Walsh BC, Dubyak GR (2010) Extracellular osmolarity modulates G protein-coupled receptor-dependent ATP release from 1321n1 astrocytoma cells. Am J Physiol Cell Physiol 298: C386–C396. 19907018 10.1152/ajpcell.00430.2009 PMC2822496

[26] Kreda SM, Seminario-Vidal L, van Heusden CA, O’Neal W, Jones L, et al (2010) Receptorpromoted exocytosis of airway epithelial mucin granules containing a spectrum of adenine nucleotides. J Physiol 588: 2255–2267. 20421285 10.1113/jphysiol.2009.186643 PMC2911224

[27] Seminario-Vidal L, Okada SF, Sesma JI, Kreda SM, van Heusden CA, et al (2011) Rho signaling regulates pannexin 1-mediated ATP release from airway epithelia. J Biol Chem 286: 26277–26286. 21606493 10.1074/jbc.M111.260562 PMC3143590

[28] Goedecke S, Roderigo C, Rose CR, Rauch BH, Goedecke A, et al (2011) Thrombin-induced ATP release from human umbilical vein endothelial cells. Am J Physiol Cell Physiol 0. 10.1152/ajpcell.00283.2010 22159088

[29] Derangeon M, Bourmeyster N, Plaisance I, Pinet-Charvet C, Chen Q, et al (2008) RhoA GTPase and F-actin dynamically regulate the permeability of Cx43-made channels in rat cardiac myocytes. J Biol Chem 283: 30754–30765. 18667438 10.1074/jbc.M801556200 PMC2662158

[30] Ponsaerts R, Wang N, Himpens B, Leybaert L, Bultynck G (2012) The contractile system as a negative regulator of the connexin 43 hemichannel. Biol Cell Epub. 10.1111/boc.201100079 22375941

[31] Anderson SC, Stone C, Tkach L, SundarRaj N (2002) Rho and Rho-kinase (ROCK) signaling in adherens and gap junction assembly in corneal epithelium. Invest Ophthalmol Vis Sci 43: 978–986. 11923237

[32] van Zeijl L, Ponsioen B, Giepmans BNG, Ariaens A, Postma FR, et al (2007) Regulation of connexin 43 gap junctional communication by phosphatidylinositol 4,5-bisphosphate. J Cell Biol 177: 881–891. 17535964 10.1083/jcb.200610144 PMC2064287

[33] Baker SM, Kim N, Gumpert AM, Segretain D, Falk MM (2008) Acute internalization of gap junctions in vascular endothelial cells in response to in–ammatory mediator-induced G-protein coupled receptor activation. FEBS Lett 582: 4039–4046. 18992245 10.1016/j.febslet.2008.10.043 PMC2628571

[34] Iyer S, Deutsch K, Yan X, Lin B (2007) Batch RNAi selector: a standalone program to predict specific siRNA candidates in batches with enhanced sensitivity. Comput Methods Programs Biomed 85: 203–209. 17197051 10.1016/j.cmpb.2006.11.004

